# Editorial: Epigenetic Therapy With Histone Deacetylase Inhibitors: Implications for Cancer Treatment

**DOI:** 10.3389/fcell.2021.662761

**Published:** 2021-04-06

**Authors:** Angela Sousa, Christiane Pienna Soares, Jean Leandro Dos Santos

**Affiliations:** ^1^CICS-UBI – Health Science Research Centre, University of Beira Interior, Covilhã, Portugal; ^2^Clinical Analysis Department, School of Pharmaceutical Sciences, São Paulo State University, Araraquara, Brazil; ^3^Drugs and Medicines Department, School of Pharmaceutical Science, São Paulo State University, Araraquara, Brazil

**Keywords:** cancer treatment, cellular mechanisms, drugs, epigenetics, histone deacetylase inhibitors, molecular biology

Epigenetic modifications including acetylation, methylation, phosphorylation, and ubiquitination, play a pivotal role in gene expression regulation. Among the enzymes involved in epigenetic modifications, histone deacetylase (HDAC) is known by promoting chromatin relaxation and gene transcription. Several HDAC isoenzymes are overexpressed in a variety of malignancies and their inhibition is a validate approach. HDAC inhibitors (HDACi) induce cancer cell cycle arrest, differentiation and death, and reduce angiogenesis and other cellular events (Glozak and Seto, 2007; Dawson and Kouzarides, 2012). Therefore, in the Research Topic “Epigenetic Therapy with Histone Deacetylase Inhibitors: Implications for Cancer Treatment,” several articles are presented to highlight exciting recent advances and provide more focused in-depth insights in several emerging areas in this field.

Li et al. described the diverse structures of HDACs and their underlying biological functions, highlighting the HDAC biological mechanisms and the potential avenues to HDACi as novel precise cancer treatments. Perla et al. discussed how HDACis display antitumor effects in experimental models of specific pediatric brain tumor types and new clinical perspectives for the use of HDACis in the treatment of these tumors. Colorectal cancer (CRC) progression is affected by both genetic and epigenetic regulations. Yeh et al. used a database mining method to evaluate a protein-protein interaction network and a candidate gene regulatory network for designing multiple-molecule drugs to prevent the progression of CRC. Freitas et al. described the role of HDACi as a possible intervention in the cervical cancer treatment induced by HPV, the major risk factor of this cancer. Yeon et al. have described in their review article the role of HDACi used alone and in combination with other drugs to overcome resistance in metastatic melanoma. Hontecillas-Prieto et al. described the limitations of monotherapy using HDACi, which was not observed for combinatorial regimens that could be explored in preclinical and clinical studies.

Based on cell MAP-kinase signaling pathways associated to prostate cancer, Corno et al. verified the effectiveness of the HDAC6 inhibitor and the MEK-inhibitor drugs in prostate cancer models and considered that HDACi can reactivate the expression of genes favoring cell response to drugs. Hu et al. studied a possible signaling pathway and molecular mechanisms by which HDAC 1 and 3 epigenetically suppressed a specific transcription factor during the epithelial—mesenchymal transition in liver cancer. Chen C.Y. et al. describes the anticancer effect of 2-*O*-methylmagnolol by *in vitro* and *in vivo* studies, which exhibited activity against hepatocellular carcinoma associated with HDAC1 inhibition. Kulka et al. gave an overview of the impact of HDACi treatment on protein quality control systems and its relationship with some cellular events associated with malignant diseases. Chen I.C. et al. describes the potential use of HDACi in clinical studies to treat T-cell and B-cell lymphomas, reporting the effectiveness and toxicity through different therapeutic approaches. Peters et al. also determined the synergism of an approved HDACi compound and drugs that can block DNA synthesis and induce DNA strand break; this combination increased the anticancer effect against lymphoma cell lines. Iannelli et al. evaluated the antitumor effects of a combination of HDACi and conventional chemotherapy drug, demonstrating antitumor effect also *in vivo* head and neck squamous cell carcinoma models. Xu et al. verified that a new HDACi hybrid improved the antitumor effect, studying *in vitro* and *in vivo* assays for potential breast cancer therapy. Mamdani and Jalal reviewed the available preclinical and clinical evidence for the use of HDACi in non-small cell lung cancer (NSCLC), emphasizing the potential efficacy of HDACi in combination with the treatment of NSCLC driven to immune checkpoint inhibitors and tyrosine kinase inhibitors. Tu et al. summarized the advances of HDACi nanomedicines to enhance HDACi therapy efficacy, discussing tumor-targeting delivery and how to achieve the site-specific controlled drug release.

Overall, the Research Topic presented here, containing original research and review of articles, highlights some current advances regarding the epigenetic therapy using HDACi, mainly focusing on cancer treatment. The topic aims to encourage scientific efforts to find new safe and effective therapies using HDACi.

## Author Contributions

All authors listed have made a substantial, direct and intellectual contribution to the work, and approved it for publication.

## Conflict of Interest

The authors declare that the research was conducted in the absence of any commercial or financial relationships that could be construed as a potential conflict of interest.
